# Supplementary material for: Elective and nonelective cesarean section and obesity among young adult male offspring: A Swedish population–based cohort study
Source: PLoS Med. 2019 Dec 6;16(12):e1002996. doi: 10.1371/journal.pmed.1002996 (PMC6897402; doi:10.1371/journal.pmed.1002996)
Supplement: S1 Table — (DOCX) [file pmed.1002996.s001.docx]

| **S1 Table. Descriptive characteristics of the total population, eligible population and the step-by-step excluded population.** | | | | | | | |
| --- | --- | --- | --- | --- | --- | --- | --- |
|  | *Excluded at conscription* | | |  | *Excluded from analysis* | | |
|  | **Conscripted (*N* = 229,632)** | **Not conscripted (*N* = 53,742)** | ***p*** |  | **Incomplete records^a^ (*N* = 132,341)** | **Analytic sample (*N* = 97,291)** | ***p*** |
| **Offspring characteristics** | | | |  |  |  |  |
| BMI category at conscription, No. (%) | | |  |  |  |  | 0.004 |
| Underweight | 9,326 (6.1) | N/A |  |  | 3,381 (6.0) | 5,945 (6.1) |  |
| Normal weight | 112,635 (73.5) | N/A |  |  | 41,124 (73.5) | 71,511 (73.5) |  |
| Overweight | 23,496 (15.3) | N/A |  |  | 8,455 (15.1) | 15,041 (15.5) |  |
| Obese | 7,765 (5.1) | N/A |  |  | 2,971 (5.3) | 4,794 (4.9) |  |
| Missing^b^ | 76,410 (33.3) | 53,742 (100.0) |  |  | 76,410 (57.7) | N/A |  |
| BMI (kg/m^2^), mean (SD) | 22.9 (3.7) (n=153,222) | N/A |  |  | 22.9 (3.8) (n=55,931) | 22.8 (3.7) | 0.100 |
| Birth weight (grams), mean (SD) | 3,590.5 (534.8) (n=226,523) | 3,518.4 (590.3) (n=52,732) | <0.001 |  | 3,571.5 (546.2) (n=129,232) | 3,615.9 (518.3) | <0.001 |
| Weeks of gestation, mean (SD) | 39.4 (1.7) (n=227,168) | 39.2 (2.0) (n=52,896) | <0.001 |  | 39.4 (1.8) (n=129,877) | 39.5 (1.6) | <0.001 |
| **Maternal characteristics** | | | |  |  |  |  |
| Maternal age at birth (years), mean (SD) | 28.4 (5.0) (n=229,631) | 28.3 (5.3) (n=53,728) | <0.001 |  | 28.4 (5.1) (n=132,340) | 28.5 (4.9) | <0.001 |
| Maternal pre-pregnancy BMI, mean (SD) | 21.9 (3.1) (n=163,518) | 22.1 (3.3) (n=37,759) | <0.001 |  | 22.0 (3.1) (n=66,227) | 21.9 (3.0) | 0.005 |
| Maternal BMI category, No. (%) | | | <0.001 |  |  |  | <0.001 |
| Underweight | 13,143 (8.0) | 3,312 (8.8) |  |  | 5,693 (8.6) | 7,450 (7.7) |  |
| Normal weight | 128,238 (78.4) | 28,390 (75.2) |  |  | 51,158 (77.2) | 77,080 (79.2) |  |
| Overweight | 18,821 (11.5) | 4,967 (13.2) |  |  | 7,888 (11.9) | 10,933 (11.2) |  |
| Obese | 3,316 (2.0) | 1,090 (2.9) | |  | 1,488 (2.2) | 1,828 (1.9) |  |
| Missing^b^ | 66,114 (28.8) | 15,983 (29.7) |  |  | 66,114 (50.0) | N/A |  |
| Parity, median (IQR) | 2.0 (1.0, 2.0) (n=228,238) | 2.0 (1.0, 3.0) (n=53,413) | <0.001 |  | 2.0 (1.0, 2.0) (n=130,947) | 2.0 (1.0, 2.0) | <0.001 |
| Maternal diabetes mellitus, No. (%) | 1,423 (0.6) | 396 (0.7) | 0.002 |  | 960 (0.7) | 463 (0.5) | <0.001 |
| Maternal hypertension, No. (%) | 542 (0.2) | 163 (0.3) | 0.005 |  | 338 (0.3) | 204 (0.2) | 0.019 |
| Preeclampsia, No. (%) | 3,551 (1.5) | 964 (1.8) | <0.001 |  | 2,036 (1.5) | 1,515 (1.6) | 0.720 |
| Maternal smoking at the commencement of pregnancy, No. (%) | | | <0.001 |  |  |  | <0.001 |
| Not smoking | 139,737 (70.8) | 31,790 (67.1) |  |  | 68,923 (68.8) | 70,814 (72.8) |  |
| 1-9 cig/day | 35,620 (18.0) | 9,098 (19.2) |  |  | 18,943 (18.9) | 16,677 (17.1) |  |
| ≥10 cig/day | 22,122 (11.2) | 6,459 (13.6) | |  | 12,322 (12.3) | 9,800 (10.1) |  |
| Missing^b^ | 32,153 (14.0) | 6,395 (11.9) |  |  | 32,153 (24.3) | N/A |  |
| **Socioeconomic factor** | | |  |  |  |  |  |
| Highest parental education, No. (%) | | | <0.001 |  |  |  | <0.001 |
| Primary education | 23,941 (10.5) | 8,280 (16.4) |  |  | 15,525 (11.9) | 8,416 (8.7) |  |
| Secondary education | 116,006 (51.0) | 27,169 (53.9) |  |  | 67,497 (51.9) | 48,509 (49.9) |  |
| University education | 87,304 (38.4) | 14,962 (29.7) | |  | 46,938 (36.1) | 40,366 (41.5) |  |
| Missing^b^ | 2,381 (1.0) | 3,331 (6.2) |  |  | 2,381 (1.8) | N/A |  |
| n indicated where fewer than N individuals had available information | | | | | | | |
| ^a^ incomplete records of body mass index (BMI), confounders or extreme values at conscription. | | | | | | | |
| ^b^ Proportion (%) of N | | | | | | | |
| Abbreviations: BMI, body mass index; cig, cigarette; IQR, interquartile range; No., number; SD, standard deviation | | | | | | | |
