# Supplementary material for: Elective and nonelective cesarean section and obesity among young adult male offspring: A Swedish population–based cohort study
Source: PLoS Med. 2019 Dec 6;16(12):e1002996. doi: 10.1371/journal.pmed.1002996 (PMC6897402; doi:10.1371/journal.pmed.1002996)
Supplement: S2 Table — (DOCX) [file pmed.1002996.s002.docx]

| **S2 Table. Relative risk ratios associated with instrumental vaginal delivery, elective cesarean section, and nonelective cesarean section as compared with vaginal delivery of underweight, overweight, and obesity relative to normal weight.** | | | | | | | | |
| --- | --- | --- | --- | --- | --- | --- | --- | --- |
|  |  | **Crude** | | |  | **Adjusted^a^** | | |
|  | **Cases, No. (%)** | **RRR** | **95% CI** | ***p*** |  | **RRR** | **95% CI** | ***p*** |
| **Underweight versus normal weight** | | | | | | | |  |
| *Vaginal* | 5,083 (6.24) | 1 | - | - |  | 1 | - | - |
| *Vaginal instrumental* | 408 (5.35) | 0.86 | 0.77–0.95 | 0.004 |  | 0.90 | 0.80–1.00 | 0.048 |
| *Elective cesarean section* | 224 (5.40) | 0.87 | 0.75–1.00 | 0.043 |  | 0.87 | 0.76–1.01 | 0.065 |
| *Nonelective cesarean section* | 230 (5.58) | 0.91 | 0.79–1.04 | 0.162 |  | 0.93 | 0.81–1.07 | 0.286 |
| **Overweight versus normal weight** |  |  |  |  |  |  |  |  |
| *Vaginal* | 12,503 (15.36) | 1 | - | - |  | 1 | - | - |
| *Vaginal instrumental* | 1,217 (15.97) | 1.04 | 0.98–1.11 | 0.227 |  | 1.02 | 0.95–1.09 | 0.608 |
| *Elective cesarean section* | 650 (15.67) | 1.02 | 0.94–1.12 | 0.613 |  | 0.99 | 0.90–1.08 | 0.839 |
| *Nonelective cesarean section* | 671 (16.29) | 1.08 | 0.99–1.17 | 0.097 |  | 0.99 | 0.90–1.08 | 0.800 |
| **Obese versus normal weight** |  |  |  |  |  |  |  |  |
| *Vaginal* | 3,938 (4.84) | 1 | - | - |  | 1 | - | - |
| *Vaginal instrumental* | 396 (5.20) | 1.08 | 0.97–1.20 | 0.183 |  | 1.02 | 0.91–1.14 | 0.729 |
| *Elective cesarean section* | 229 (5.52) | 1.14 | 1.00–1.31 | 0.056 |  | 1.02 | 0.88–1.18 | 0.812 |
| *Nonelective cesarean section* | 231 (5.61) | 1.18 | 1.02–1.35 | 0.021 |  | 0.96 | 0.83–1.10 | 0.555 |
| Empty cells (-) indicate reference group. | | | | | | | | |
| ^a^Adjusted for: Prepregnancy maternal body mass index (BMI), maternal diabetes at delivery, maternal hypertension at delivery, maternal smoking, parity, parental education, maternal age at delivery, birth weight standardized according to gestational age, preeclampsia and gestational age | | | | | | | | |
| Abbreviations: CI, confidence interval; No., number; RRR, relative risk ratio. | | | | | | | | |
