# Supplementary material for: Elective and nonelective cesarean section and obesity among young adult male offspring: A Swedish population–based cohort study
Source: PLoS Med. 2019 Dec 6;16(12):e1002996. doi: 10.1371/journal.pmed.1002996 (PMC6897402; doi:10.1371/journal.pmed.1002996)
Supplement: S3 Table — (DOCX) [file pmed.1002996.s003.docx]

| **S3 Table. Sensitivity analysis on associations between elective and nonelective cesarean section as compared with vaginal delivery of underweight, overweight, and obesity relative to normal weight.** | | | | | | | | | | | | | | | | | | | |
| --- | --- | --- | --- | --- | --- | --- | --- | --- | --- | --- | --- | --- | --- | --- | --- | --- | --- | --- | --- |
|  | **Model 1 (*N* = 97,291)** | | |  | **Model 2 (*N* = 96,050)** | | |  | **Model 3 (*N* = 97,291)** | | |  | **Model 4 (*N* = 97,353)** | | |  | **Model 5 (*N* = 97,291)** | | |
|  | **RRR** | **95% CI** | ***p*** |  | **RRR** | **95% CI** | ***p*** |  | **RRR** | **95% CI** | ***p*** |  | **RRR** | **95% CI** | ***p*** |  | **RRR** | **95% CI** | ***p*** |
| **Underweight versus normal weight** | | |  |  |  |  |  |  |  |  |  |  |  |  |  |  |  |  |  |
| *Vaginal* | 1 | - | - |  | 1 | - | - |  | 1 | - | - |  | 1 | - | - |  | 1 | - | - |
| *Elective cesarean section* | 0.87 | 0.76–1.01 | 0.063 |  | 0.87 | 0.76–1.01 | 0.066 |  | 0.90 | 0.78–1.05 | 0.175 |  | 0.88 | 0.76–1.01 | 0.072 |  | 0.88 | 0.76–1.01 | 0.071 |
| *Nonelective cesarean section* | 0.94 | 0.81–1.08 | 0.350 |  | 0.94 | 0.82–1.09 | 0.432 |  | 0.94 | 0.82–1.08 | 0.407 |  | 0.94 | 0.81–1.08 | 0.358 |  | 0.92 | 0.80–1.06 | 0.272 |
| **Overweight versus normal weight** | | |  |  |  |  |  |  |  |  |  |  |  |  |  |  |  |  |  |
| *Vaginal* | 1 | - | - |  | 1 | - | - |  | 1 | - | - |  | 1 | - | - |  | 1 | - | - |
| *Elective cesarean section* | 0.99 | 0.90–1.08 | 0.790 |  | 0.97 | 0.89–1.06 | 0.542 |  | 0.97 | 0.88–1.07 | 0.581 |  | 0.99 | 0.90–1.08 | 0.817 |  | 0.98 | 0.89–1.07 | 0.592 |
| *Nonelective cesarean section* | 0.99 | 0.90–1.08 | 0.755 |  | 0.97 | 0.88–1.06 | 0.443 |  | 0.98 | 0.90–1.07 | 0.707 |  | 0.99 | 0.90–1.08 | 0.767 |  | 0.98 | 0.90–1.08 | 0.706 |
| **Obese versus normal weight** | | |  |  |  |  |  |  |  |  |  |  |  |  |  |  |  |  |  |
| *Vaginal* | 1 | - | - |  | 1 | - | - |  | 1 | - | - |  | 1 | - | - |  | 1 | - | - |
| *Elective cesarean section* | 1.01 | 0.88–1.17 | 0.859 |  | 0.99 | 0.85–1.15 | 0.895 |  | 1.05 | 0.90–1.22 | 0.565 |  | 1.01 | 0.87–1.17 | 0.895 |  | 1.01 | 0.87–1.17 | 0.930 |
| *Nonelective cesarean section* | 0.95 | 0.82–1.09 | 0.465 |  | 0.93 | 0.81–1.08 | 0.346 |  | 0.96 | 0.83–1.11 | 0.587 |  | 0.95 | 0.82–1.09 | 0.476 |  | 0.94 | 0.82–1.09 | 0.439 |
| Empty cells (-) indicate reference group. | | | | | | | | | | | | | | | | | | | |
| Model 1: Adjusted for confounders + Cubic transformed maternal age and maternal body mass index. | | | | | | | | | | | | | | | | | | | |
| Model 2: Adjusted for confounders + Gestational weight gain z-score | | | | | | | | | | | | | | | | | | | |
| Model 3: Adjusted for confounders + Previous cesarean delivery. | | | | | | | | | | | | | | | | | | | |
| Model 4: Adjusted for confounders + including those with extreme values excluded from analytical cohort. | | | | | | | | | | | | | | | | | | | |
| Model 5: Adjusted for confounders + Relaxed linearity of maternal age, maternal BMI, gestational age and birth weight standardized according to gestational age, using restricted cubic splines with 5 knots at: 5 27.5 50 72.5 95 percentiles. | | | | | | | | | | | | | | | | | | | |
| Abbreviations: CI, confidence interval; RRR, relative risk ratio. | | | | | | | | | | | | | | | | | | | |
