# Supplementary material for: Elective and nonelective cesarean section and obesity among young adult male offspring: A Swedish population–based cohort study
Source: PLoS Med. 2019 Dec 6;16(12):e1002996. doi: 10.1371/journal.pmed.1002996 (PMC6897402; doi:10.1371/journal.pmed.1002996)
Supplement: S4 Table — (DOCX) [file pmed.1002996.s004.docx]

| **S4 Table. Association between pooled cesarean section and underweight, overweight, and obesity as compared with normal weight.** | | | | | | | | |
| --- | --- | --- | --- | --- | --- | --- | --- | --- |
|  |  | **Crude** | | |  | **Adjusted^a^** | | |
|  | **Cases, No. (%)** | **RRR** | **95% CI** | ***p*** |  | **RRR** | **95% CI** | ***p*** |
| **Underweight versus normal weight** | | | |  |  |  |  |  |
| *Vaginal* | 5,491 (6.17) | 1 | - | - |  | 1 | - | - |
| *Cesarean section* | 454 (5.49) | 0.90 | 0.81–0.99 | 0.033 |  | 0.91 | 0.82–1.01 | 0.066 |
| **Overweight versus normal weight** | | | |  |  |  |  |  |
| *Vaginal* | 13,720 (15.41) | 1 | - | - |  | 1 | - | - |
| *Cesarean section* | 1,321 (15.98) | 1.05 | 0.98–1.11 | 0.165 |  | 0.99 | 0.93–1.05 | 0.716 |
| **Obese versus Normal Weight** | | | |  |  |  |  |  |
| *Vaginal* | 4,334 (4.87) | 1 | - | - |  | 1 | - | - |
| *Cesarean section* | 460 (5.56) | 1.15 | 1.04–1.27 | 0.005 |  | 0.98 | 0.89–1.09 | 0.775 |
| Empty cells (-) indicate reference group. | | | | | | | | |
| ^a^Adjusted for: Prepregnancy maternal body mass index (BMI), maternal diabetes at delivery, maternal hypertension at delivery, maternal smoking, parity, parental education, maternal age at delivery, birth weight standardized according to gestational age, preeclampsia and gestational age. | | | | | | | | |
| Abbreviations: CI, confidence interval; No., number; RRR, relative risk ratio. | | | | | | | | |
