# Supplementary material for: Elective and nonelective cesarean section and obesity among young adult male offspring: A Swedish population–based cohort study
Source: PLoS Med. 2019 Dec 6;16(12):e1002996. doi: 10.1371/journal.pmed.1002996 (PMC6897402; doi:10.1371/journal.pmed.1002996)
Supplement: S8 Table — (DOCX) [file pmed.1002996.s008.docx]

| **S8 Table. Fixed-effects linear association between mode of delivery and continuous body mass index in a subset of full brothers (N= 9,676).** | | | | | | | | |
| --- | --- | --- | --- | --- | --- | --- | --- | --- |
|  |  | **Crude** | | |  | **Adjusted^a^** | | |
|  | **Exposed, No. (%)** | **Mean difference** | **95% CI** | ***p*** |  | **Mean difference** | **95% CI** | ***p*** |
| *Vaginal* | 9,032 (93.34) | Ref. | - | - |  | Ref. | - | - |
| *Elective cesarean section* | 330 (3.41) | 0.19 | -0.40–0.78 | 0.524 |  | 0.21 | -0.39–0.80 | 0.494 |
| *Nonelective cesarean section* | 314 (3.25) | 0.16 | -0.38–0.70 | 0.557 |  | 0.11 | -0.43–0.64 | 0.701 |
| Empty cells (-) indicate reference group. | | | | | | | | |
| ^a^Adjusted for: Pre-pregnancy maternal body mass index (BMI), maternal diabetes at delivery, maternal hypertension at delivery, maternal smoking, parity, maternal age at delivery, birth weight standardized according to gestational age, preeclampsia and gestational age. | | | | | | | | |
| Abbreviations: CI, confidence interval; No., number; Ref., reference. | | | | | | | | |
