# Supplementary material for: Elective and nonelective cesarean section and obesity among young adult male offspring: A Swedish population–based cohort study
Source: PLoS Med. 2019 Dec 6;16(12):e1002996. doi: 10.1371/journal.pmed.1002996 (PMC6897402; doi:10.1371/journal.pmed.1002996)
Supplement: S9 Table — (DOCX) [file pmed.1002996.s009.docx]

| **S9 Table. Association between mode of delivery and underweight, overweight, and obesity as compared with normal weight, excluding those born before 37 weeks of gestation (preterm), restricting to those born between 37 weeks and 41 weeks and 6 days of gestation (at term), and not adjusting for gestational age.** | | | | | | | | | | | | | | | | |
| --- | --- | --- | --- | --- | --- | --- | --- | --- | --- | --- | --- | --- | --- | --- | --- | --- |
|  | **Main analysis^a^ (*N* = 97,291)** | | |  | **Excluding those born preterm ^a^**  **(*N* = 88,227)** | | |  | **Restricted to those born at term^a^ (*N* = 87,541)** | | |  | **Not adjusting for gestational age^b^ (N = 97,291)** | | |  |
|  | **RRR** | **95% CI** | ***p*** |  | **RRR** | **95% CI** | ***p*** |  | **RRR** | **95% CI** | ***p*** |  | **RRR** | **95% CI** | ***p*** |  |
| **Underweight versus normal weight** | | |  |  |  |  |  |  |  |  |  |  |  |  |  |  |
| *Vaginal* | 1 | - | - |  | 1 | - | - |  | 1 | - | - |  | 1 | - | - |  |
| *Elective cesarean section* | 0.88 | 0.76–1.01 | 0.079 |  | 0.88 | 0.75–1.03 | 0.114 |  | 0.86 | 0.74–1.01 | 0.062 |  | 0.87 | 0.76–1.00 | 0.053 |  |
| *Nonelective cesarean section* | 0.97 | 0.81–1.08 | 0.359 |  | 0.92 | 0.78–1.08 | 0.330 |  | 0.93 | 0.79–1.10 | 0.391 |  | 0.93 | 0.81–1.07 | 0.313 |  |
| **Overweight versus normal weight** | | |  |  |  |  |  |  |  |  |  |  |  |  |  |  |
| *Vaginal* | 1 | - | - |  | 1 |  |  |  | 1 |  |  |  | 1 | - | - |  |
| *Elective cesarean section* | 0.99 | 0.90–1.08 | 0.818 |  | 1.00 | 0.94–1.14 | 0.53 |  | 1.02 | 0.93–1.13 | 0.657 |  | 0.99 | 0.91–1.08 | 0.830 |  |
| *Nonelective cesarean section* | 0.99 | 0.90–1.08 | 0.764 |  | 1.06 | 0.96–1.17 | 0.25 |  | 1.07 | 0.96–1.18 | 0.212 |  | 0.99 | 0.90–1.08 | 0.771 |  |
| **Obese versus normal weight** | | |  |  |  |  |  |  |  |  |  |  |  |  |  |  |
| *Vaginal* | 1 | - | - |  | 1 | - | - |  | 1 | - | - |  | 1 | - | - |  |
| *Elective cesarean section* | 1.02 | 0.88–1.18 | 0.826 |  | 1.07 | 0.91–1.26 | 0.39 |  | 1.06 | 0.91–1.25 | 0.453 |  | 1.05 | 0.91–1.21 | 0.535 |  |
| *Nonelective cesarean section* | 0.96 | 0.83–1.10 | 0.532 |  | 1.01 | 0.86–1.18 | 0.92 |  | 1.01 | 0.86–1.18 | 0.936 |  | 0.97 | 0.84–1.11 | 0.633 |  |
| Empty cells (-) indicate reference group. | | | | | | | | | | | | | | | | |
| ^a^Adjusted for: Prepregnancy maternal BMI, maternal diabetes at delivery, maternal hypertension at delivery, maternal smoking, parity, parental education, maternal age at delivery, birth weight standardized according to gestational age, preeclampsia and gestational age. | | | | | | | | | | | | | | | | |
| ^b^Adjusted for: same as above, excluding gestational age. | | | | | | | | | | | | | | | | |
| Abbreviations: BMI, body mass index; CI, confidence interval; RRR, relative risk ratio | | | | | | | | | | | | | | | | |
