# Supplementary material for: Elective and nonelective cesarean section and obesity among young adult male offspring: A Swedish population–based cohort study
Source: PLoS Med. 2019 Dec 6;16(12):e1002996. doi: 10.1371/journal.pmed.1002996 (PMC6897402; doi:10.1371/journal.pmed.1002996)
Supplement: S10 Table — (DOCX) [file pmed.1002996.s010.docx]

| **S10 Table. Crude and adjusted association between mode of delivery and underweight, overweight, and obesity as compared with normal weight using generalized ordered logit estimation.** | | | | | | | |
| --- | --- | --- | --- | --- | --- | --- | --- |
|  | **Crude**  **(*N* = 97,291)** | | |  | **Adjusted^a^**  **(*N* = 97,291)** | | |
|  | **OR** | **95% CI** | ***p*** |  | **OR** | **95% CI** | ***p*** |
| **Underweight to normal weight** | | |  |  |  |  |  |
| *Vaginal* | 1 | - | - |  | 1 | - | - |
| *Elective cesarean section* | 1.15 | 1.00–1.32 | 0.045 |  | 1.13 | 0.98–1.30 | 0.095 |
| *Nonelective cesarean section* | 1.11 | 0.97–1.27 | 0.126 |  | 1.07 | 0.93–1.23 | 0.331 |
| **Normal weight to overweight** | | |  |  |  |  |  |
| *Vaginal* | 1 | - | - |  | 1 | - | - |
| *Elective cesarean section* | 1.06 | 0.98–1.14 | 0.152 |  | 1.00 | 0.93–1.09 | 0.920 |
| *Nonelective cesarean section* | 1.10 | 1.02–1.19 | 0.012 |  | 0.99 | 0.91–1.07 | 0.721 |
| **Overweight to obese** | | |  |  |  |  |  |
| *Vaginal* | 1 | - | - |  | 1 | - | - |
| *Elective cesarean section* | 1.14 | 1.00–1.31 | 0.057 |  | 1.04 | 0.90–1.20 | 0.571 |
| *Nonelective cesarean section* | 1.16 | 1.01–1.33 | 0.032 |  | 0.97 | 0.84–1.11 | 0.649 |
| Empty cells (-) indicate reference group. | | | | | | | |
| ^a^Adjusted for: Prepregnancy maternal BMI, maternal diabetes at delivery, maternal hypertension at delivery, maternal smoking, parity, parental education, maternal age at delivery, birth weight standardized according to gestational age, preeclampsia and gestational age. | | | | | | | |
| Abbreviations: CI, confidence interval; OR, odds ratio. | | | | | | | |
