# Supplementary material for: Elective and nonelective cesarean section and obesity among young adult male offspring: A Swedish population–based cohort study
Source: PLoS Med. 2019 Dec 6;16(12):e1002996. doi: 10.1371/journal.pmed.1002996 (PMC6897402; doi:10.1371/journal.pmed.1002996)
Supplement: S11 Table — (DOCX) [file pmed.1002996.s011.docx]

| **S11 Table. Association between mode of delivery and underweight, overweight, and obesity as compared with normal weight, only adjusting for maternal prepregnancy body mass index.** | | | | | | | |
| --- | --- | --- | --- | --- | --- | --- | --- |
|  | **Crude**  **(*N* = 97,291)** | | |  | **Only adjusting for maternal prepregnancy BMI (*N* = 97,291)** | | |
|  | **RRR** | **95% CI** | ***p*** |  | **RRR** | **95% CI** | ***p*** |
| **Underweight versus normal weight** | | |  |  |  |  |  |
| *Vaginal* | 1 | - | - |  | 1 | - | - |
| *Elective cesarean section* | 0.88 | 0.76–1.01 | 0.064 |  | 0.91 | 0.80–1.05 | 0.211 |
| *Nonelective cesarean section* | 0.92 | 0.80–1.05 | 0.220 |  | 0.97 | 0.85–1.12 | 0.686 |
| **Overweight versus normal weight** | | |  |  |  |  |  |
| *Vaginal* | 1 | - | - |  | 1 |  |  |
| *Elective cesarean section* | 1.02 | 0.93–1.11 | 0.669 |  | 0.96 | 0.88–1.05 | 0.326 |
| *Nonelective cesarean section* | 1.07 | 0.98–1.17 | 0.113 |  | 0.99 | 0.91–1.09 | 0.909 |
| **Obese versus normal weight** | | |  |  |  |  |  |
| *Vaginal* | 1 | - | - |  | 1 | - | - |
| *Elective cesarean section* | 1.14 | 0.99–1.30 | 0.069 |  | 0.99 | 0.85–1.14 | 0.840 |
| *Nonelective cesarean section* | 1.17 | 1.02–1.34 | 0.027 |  | 1.01 | 0.88–1.16 | 0.873 |
| Empty cells (-) indicate reference group. | | | | | | | |
| Abbreviations: CI, confidence interval; RRR, relative risk ratio. | | | | | | | |
