# Supplementary material for: Elective and nonelective cesarean section and obesity among young adult male offspring: A Swedish population–based cohort study
Source: PLoS Med. 2019 Dec 6;16(12):e1002996. doi: 10.1371/journal.pmed.1002996 (PMC6897402; doi:10.1371/journal.pmed.1002996)
Supplement: Attachment — Submitted filename: ahlqvist.pdf [file pmed.1002996.s012.pdf]

This paper discusses the association between Caesarian section and early male obesity in Sweden,

I was asked for a statistical report and I interpret that to include all aspects of the design and conduct of the study.

Points of detail

Page 1 I might mention Sweden in the title.

Page 2 and elsewhere The wording the authors use varies between saying they found no association and saying there was no association. What they found, as I am sure they appreciate, is no evidence of association. I think I would make the wording consistent throughout even at the cost of repeating the phrase 'we found no evidence'.

Page 2 Typo, offspring, not offspring's.

Page 4 I think we need some justification of the time period chosen for the cohort. The conscription data–set, and hence the anthropometry, dates from 2005. I know the authors mention this on page 13 (but not in the section on limitations) but why did they make this choice?

Page 5 In fact the extreme values were used in a sensitivity analysis and I would mention here, perhaps by adding the phrase "except for sensitivity analyses" otherwise the reader is going to read this and ask whether excluding them made a difference.

Page 7 The primary outcome is presented here as an ordered categorical variable (BMI) with four levels. Table 2 appears to confirm this but the text on page 10 describes the association only in terms of obesity, the most extreme category. I think this all needs clarifying as at the moment is confusing.

Page 7 If the primary outcome is indeed an ordered categorical one why not analyse it as such rather than as unordered?

Page 7 I am not sure I would have said parity was a continuous variable but in any event it was treated as categorical anyway.

Page 8 For variables which are confounders and whose association is not of primary interest it seems a good idea not to insist on linearity as mentioned here. Why then only relax this for some variables? Why not use splines which make fewer assumptions about the exact shape?

Page 10 If the authors used logistic regression then the estimates are presumably odds ratios rather than risk ratios which would have required a log–binomial model.

References It might be a kindness to the reader to give translated titles for the ones in Swedish although I suppose if you cannot understand the title you are unlikely to try to fetch the article.

Supplement This finished with eight blank pages. I suspect there was nothing missing.

Summary

Some queries and suggestions about the analysis.

Michael Dewey
