# Supplementary material for: Elective and nonelective cesarean section and obesity among young adult male offspring: A Swedish population–based cohort study
Source: PLoS Med. 2019 Dec 6;16(12):e1002996. doi: 10.1371/journal.pmed.1002996 (PMC6897402; doi:10.1371/journal.pmed.1002996)
Supplement: Attachment — Submitted filename: Reviwer comments Point-by-point version Final version.docx [file pmed.1002996.s013.docx]

**POINT-BY-POINT ADDRESSAL OF COMMENTS
-----------------------------------------------------------**

**Requests from the editors:**

**1. PLOS Medicine requires that the de-identified data underlying the specific results in a published article be made available, without restrictions on access, in a public repository or as Supporting Information at the time of article publication, provided it is legal and ethical to do so. Please see the policy at https://eur01.safelinks.protection.outlook.com/?url=http%3A%2F%2Fjournals.plos.org%2Fplosmedicine%2Fs%2Fdata-availability&amp;data=02%7C01%7Cdaniel.berglind%40ki.se%7C14ec1e8131b6495dfab608d745f39c27%7Cbff7eef1cf4b4f32be3da1dda043c05d%7C0%7C0%7C637054785399004990&amp;sdata=mO16U9wo%2Btg8Yx8tVEsqWmWSElfImJoxq29qC1K5i5A%3D&amp;reserved=0**

**and FAQs at https://eur01.safelinks.protection.outlook.com/?url=http%3A%2F%2Fjournals.plos.org%2Fplosmedicine%2Fs%2Fdata-availability%23loc-faqs-for-data-policy&amp;data=02%7C01%7Cdaniel.berglind%40ki.se%7C14ec1e8131b6495dfab608d745f39c27%7Cbff7eef1cf4b4f32be3da1dda043c05d%7C0%7C0%7C637054785399004990&amp;sdata=mk77OJO%2FvC%2FoGuHZvFgkv%2FVpg%2BUw8ixpFmI2vBqCAdM%3D&amp;reserved=0**

**Specifically, please provide a URL or email contact information from where data can be requested. Please note that the contact for data access cannot be one of the authors.**

**Answer:** As we described in the Data availability section, we will not be able to make data publicly available. We have removed the paragraph, and clarified that Swedish law prohibits such sharing and where readers may seek more information to make data-related inquiries (Page 18, Line 376):”Swedish secrecy law prohibits us from making register data publicly available. The data supporting our findings were used under license and ethical approval for the current study. Readers interested in obtaining microdata or replicating our study may seek similar approvals and inquire through Statistics Sweden. For further advice see: <https://www.scb.se/en/services/guidance-for-researchers-and-universities/>.”

**2. Title: Please include that the study was based on a Swedish population. Also, we suggest that you please change “risk for” to “association with” in the title.**

**Answer:** We appreciate this feedback, and have now changed the title to: “Elective and non-elective cesarean section and the association with obesity among young adult males: a Swedish population-based cohort study”

**3. Abstract: Please include the years during which the study took place, and length of follow up. Specifically, please include the relevant information on cohort birth years, and year of conscription.**

**Answer:**

This is also valuable feedback, and have now clarified this in the abstract (Page 2, Line 25): “Using Swedish population registers, a cohort of 97,291 males born between 1982 and 1987 were followed from birth until conscription (median 18 years of age) if they conscripted before 2006”

Please note that we sampled males who were conscripted before 2006, not before 2005 as previously stated.

**4. Abstract: Please quantify the main results (with 95% CIs and p values). Specifically, please include p values for tests of association between C-section birth and obesity.**

**Answer:** We now present 95% CIs and *p* values for all associations described in the abstract.

**5. Abstract: Please include the specific dependent variables that are adjusted for in the adjusted analyses described.**

**Answer:** We have clarified this (Page 2, Line 36) as: “… after accounting for pre-pregnancy maternal BMI, maternal diabetes at delivery, maternal hypertension at delivery, maternal smoking, parity, parental education, maternal age at delivery, gestational age, birth weight standardized according to gestational age, and preeclampsia.”

**6. Abstract: Please address the study implications without overreaching what can be concluded from the data; your study is observational and therefore causality cannot be inferred from your results. Please remove language that implies causality, such as “This suggests that there is no clinically relevant causal role of CS in the development of obesity and that an association between CS and offspring obesity may be a function of confounding.”**

**Answer:** We completely concur, and have reworded the conclusions in the Abstract as well as in the Discussion sections (Page 2, Line 45 and Page 18, Line 358, respectively) as: “This suggests that there is no clinically relevant association between CS and the development of obesity.”

Further, in accordance with concern raised regarding the discussion section (specifically Editors’ comment 13), we have now removed the speculation that previous reports of an association between CS and obesity may be a function of confounding.

**7. At this stage, we ask that you include a short, non-technical Author Summary of your research to make findings accessible to a wide audience that includes both scientists and non-scientists. The Author Summary should immediately follow the Abstract in your revised manuscript. This text is subject to editorial change and should be distinct from the scientific abstract. Please see our author guidelines for more information:** [**https://eur01.safelinks.protection.outlook.com/?url=https%3A%2F%2Fjournals.plos.org%2Fplosmedicine%2Fs%2Frevising-your-manuscript%23loc-author-summary&amp;data=02%7C01%7Cdaniel.berglind%40ki.se%7C14ec1e8131b6495dfab608d745f39c27%7Cbff7eef1cf4b4f32be3da1dda043c05d%7C0%7C0%7C637054785399004990&amp;sdata=s95%2B2%2FeGkSwES0%2BmCuolQdzSV657A8n6o%2BrQOSAOGUY%3D&amp;reserved=0**](https://eur01.safelinks.protection.outlook.com/?url=https%3A%2F%2Fjournals.plos.org%2Fplosmedicine%2Fs%2Frevising-your-manuscript%23loc-author-summary&amp;data=02%7C01%7Cdaniel.berglind%40ki.se%7C14ec1e8131b6495dfab608d745f39c27%7Cbff7eef1cf4b4f32be3da1dda043c05d%7C0%7C0%7C637054785399004990&amp;sdata=s95%2B2%2FeGkSwES0%2BmCuolQdzSV657A8n6o%2BrQOSAOGUY%3D&amp;reserved=0)

**Answer:**

**We have now included the following Author Summary immediately following the abstract** (Page 3, Line 45):

**Why Was This Done?**

- **The global prevalence of cesarean deliveries is increasing, and clarification of any harmful consequences for offspring health is warranted.**
- There is evidence of an association between delivery by cesarean section and obesity, but it may be driven by unmeasured confounding.
- **It has been suggested that elective but not non-elective cesarean section is associated with offspring obesity, but the knowledge base to support this notion is scant.**

**What Did the Researchers Do and Find?**

**A register-based total population cohort of 97,291 Swedish males were followed-up for levels of overweight and obesity in early adulthood, using objective measures of weight and height at military conscription. Sibling-comparison was used to address bias from familial factors.**

- **Delivery by cesarean section, regardless of whether elective or non-elective, was not associated with overweight or obesity among Swedish men in young adulthood.**

**What Do These Findings Mean?**

- **Mode of delivery may not be an important factor in the origins of overweight and obesity.**

**8. Methods: Please clarify the age(s) of the individuals at the time of conscription (when BMI was assessed).**

**Answer:**

We have now included a clarification of the median age at conscription in the Method section under Study population (Page 6, Line 133): “In total, 97,291 male conscripts, with a median age of 18 years at follow-up (i.e. conscription), remained eligible for analysis and were included in the final analytical sample”

We have also added the median and interquartile range of age at conscription in Table 1 (Page 11, Line 224) for further clarity.

**9. Results: Please provide 95% CIs and p values for statistical tests in support of the assertions that the prevalence of obesity varied slightly between delivery modalities (lines 176-178) and that most covariates did not vary substantially with mode of delivery (lines 178-179).**

**Answer:**

We agree that unadjusted inferential statistics may provide valuable information to readers, and now provide 95% CIs and *p* values for crude comparisons in the Results section (Page 10, Line 211) :

“The prevalence of obesity among those born by vaginal delivery was 4.9% (CI 95%: 4.7-5.0%), which was not statistically significantly different as compared to those born by elective CS (5.5%, CI 95%: 4.8-6.2, *p* = 0.057). The prevalence of obesity among those born to non-elective CS was 5.6% (CI 95%: 4.9-6.3), which was statistically significantly higher as compared to those born by vaginal delivery (*p* = 0.032). The mean BMI was lower among those born by vaginal delivery (22.8, CI 95% 22.8-22.8) as compared to those born by elective CS (23.0, CI 95%: 22.9-23.1, *p* = 0.010) or non-elective CS (23.1, CI 95%: 22.9-23.2, *p* < 0.001).”

We have now clarified that all covariates differed statistically significantly between exposure categories (Page 10, Line 217): “Although statistically different (All *p* < 0.05), most covariates did not vary substantially with mode of delivery.”

**10. Results: Line 186-187: Please revise the statement: “In our primary analysis, there was an insignificant association between elective CS and obesity (RRR 1.14, CI 95%: 0.99-1.30)...” to more accurately state that there was no significant association observed between elective CS and obesity.**

**Answer:**

We are thankful for this feedback and we have now clarified the sentence in the Results section under Mode of delivery and offspring obesity (Page 11, Line 226): “In our primary analysis, there was no statistically significant association between elective CS and obesity (RRR 1.14, CI 95%: 0.99-1.30), while a similar association between non-elective CS and obesity differed from unity (RRR 1.17, CI 95%: 1.02-1.34), as compared to vaginal delivery.”

**11. Results: Line 188: Please describe which factors are adjusted for in the analysis of the association between CS and obesity in the adjusted models.**

**Answer:**

We have now clarified this in (Page 11, Line 230) as: “We observed no association between elective nor non-elective CS and obesity (RRR 1.02 CI 95%: 0.88-1.18 & RRR 0.96, CI 95%: 0.83-1.10, respectively), as compared to vaginal delivery, when accounting for pre-pregnancy maternal BMI, maternal diabetes at delivery, maternal hypertension at delivery, maternal smoking, parity, parental education, maternal age at delivery, gestational age, birth weight standardized according to gestational age, and preeclampsia.”

**12. Results: Please provide p values for the sensitivity analyses, and tests for associations between mode of delivery and offspring obesity status.**

**Answer:** We have now provided *p* values in addition to 95% CIs throughout the text and tables.

**13. Discussion: Lines 218-220, and at 293-295: Please revise the language to avoid any misleading assertion that the lack of any association identified in the present study serves as supporting evidence for the idea that associations identified in other studies are attributable to confounding of elective vs. non-elective c-sections.**

**Answer:**

We acknowledge that the absence of an association in our study does not imply that previous studies have been confounded. We have now removed this speculation from the Discussion under Main findings (Page 15, Line 270) and Conclusion (Page 18, Line 358). Further, as this was included in the Abstract as an excerpt, we have also modified the Abstract section (Page 3, Line 46).

**14. Discussion: Lines 251-252: Please clarify where the analyses of association between CS and obesity adjusted only for pre-pregnancy BMI are presented.**

**Answer:**

These point estimates were originally provided in the Discussion section’s text, but we have now added a table in the supplement (Supplementary material: sTable 11, Page 11, Line 74) presenting RRRs, 95% CIs and *p*-values from these analyses for clarity. We now refer to this new table in the Discussion (Page 16, Line 302) as: “In our cohort, only accounting for maternal pre-pregnancy BMI attenuated the association between elective or non-elective CS and obesity by 15% and 16% respectively (RRR 1.14 vs 0.99 and 1.17 vs 1.01) (S1 Appendix: sTable 11).”

**15. Discussion: Lines 274-276: Were any statistical tests done to support the assertion that characteristics did or did not differ between the analyzed and excluded populations? If so, please present these in the results, and sTable1.**

**Answer:** We did not do such tests initially, arguing that they (as also small differences reach statistical significance in our large sample) may not help understanding of how exclusions may impact on internal and/or external validity. However, as one can argue differently, we now provide statistical tests (Chi^2^, T-test & Wilcoxon) of differences in study population characteristics between each exclusion wave. These are presented in sTable1 (Supplementary material: Page 2, Line 33) and in the text (Page 14, Line 237) as: “Characteristics in individuals who were and were not conscripted did not differ materially overall, although differences were statistically significant in our large sample (All p < 0.05, S1 Appendix: sTable 1). Similarly, although every characteristic except mean BMI and maternal preeclampsia being statistically different (*p* < 0.05), there was no major discrepancy between those with incomplete records and those retained in the analytical cohort. However, we noted that parental university education was more common among the conscripted as compared to the not conscripted (38.4% vs 29.7%, *p* < 0.05) and more common among the retained analytical cohort as compared to those with incomplete records (41.5% vs 36.1%, *p* < 0.05).”

Further, we now also clarified the above-mentioned lines the Discussion section under Strengths and limitations to highlight that differences were *statistically* different (Page 17, Line 330): “ Despite the potential for selection bias we did not observe major differences, albeit most factors being statistically significantly different (P < 0.05), between our analytic sample and the excluded populations, with the exception for highest parental education (S1 Appendix: sTable 1).”

Finally, we describe the statistical tests carried out in the Method section under Sensitivity analysis by the following sentence (Page 9, Line 181): “We descriptively present the available characteristics of the individuals not participating in conscription, and those excluded due to missing data, and compare these to the conscripted and analytic sample, respectively, using χ²-test (categorical), T-test (continuous) and Wilcoxon rank sum test (continuous skewed).”

**16. Tables: sTable7: There is a footnote for “a” but no “a” in the table. Please update footnote “b” to reflect the adjusted factors in the model.**

**Answer:** We are thankful for this observation, and have now corrected the typo in sTable 7 (Supplementary material: Page 8, Line 67).

**17. Thank you for including the STROBE checklist. Please revise the checklist to use section and paragraph numbers, rather than page numbers and line numbers.**

**Answer:** We have now revised the STROBE checklist to include section and paragraph numbers. Notably, as some aspects of our manuscripts have changed, we have revised the STROBE checklist accordingly.

**Comments from the reviewers:**

**Reviewer #1:**

**Page 1 I might mention Sweden in the title.**

**Answer:**

We agree with this point, and have, in accordance with this comment and an editorial ditto, changed the title to: “Elective and non-elective cesarean section and the association with obesity among young adult males: a Swedish population-based cohort study”

**Page 2 and elsewhere The wording the authors use varies between saying**

**they found no association and saying there was no association. What**

**they found, as I am sure they appreciate, is no evidence of association.**

**I think I would make the wording consistent throughout even at the**

**cost of repeating the phrase `we found no evidence'.**

**Answer:** We are thankful for this comment, and have changed the wording throughout the abstract to (Page 2, line 33 & line 39 & line 44): “We found no evidence of an association…”

**Page 2 Typo, offspring, not offspring's.**

**Answer:**

We have now corrected the typo in the Abstract under Conclusions (Page 3, Line 47).

**Page 4 I think we need some justification of the time period chosen for the**

**cohort. The conscription data{set, and hence the anthropometry, dates**

**from 2005. I know the authors mention this on page 13 (but not in the**

**section on limitations) but why did they make this choice?**

**Answer:**

We erroneously stated that we sampled all individuals who were conscripted before 2005, this has been corrected to before 2006.

We include individuals who were conscripted before 2006 since the Swedish conscription procedure changed after 2005. The number of conscription centers was first reduced in 2006. In 2007, a web-based screening of eligible conscripts was implemented pre-conscription, and only those meeting certain criteria (by personal traits, law abidance, and physical and psychological health) went on to be conscripted (by Swedish Defence Military Agency decree TPVFS 2007:1). Although selection bias may be a concern also in our sample recruited based on conscriptions before 2005, as argued in the Discussion section (Strengths and Limitations), we sought to minimize this bias by the restriction thus described.

To highlight this, without having to make extensive referencing to sources only available in Swedish we have now clarified this in the Method section under Study population (Page 5, Line 125): “During the study period, conscription was mandatory by law in Sweden for all male citizens up to the age of 47 (34). The study period was selected because of the changes in conscription procedures occurring after the set period. During the study period, males could only be exempt from conscriptions (requiring state approval) if they suffered from chronic disease or severe handicap (35).”

**Page 5 In fact the extreme values were used in a sensitivity analysis and I**

**would mention here, perhaps by adding the phrase "except for sensitivity analyses" otherwise the reader is going to read this and ask whether excluding them made a difference.**

**Answer:**

We appreciate this comment, as we can see how this could concern readers. We have now included a clarification in the Method section under Study population (Page 6, Line 127): “Except for in sensitivity analyses, we excluded those with extreme values of weight (≤40 or ≥150 kg), height (≤150 or ≥210 cm) and/or BMI (≤15 or ≥60 kg/m2) at conscription (n=105), in accordance with previous studies (36).”

**Page 7 The primary outcome is presented here as an ordered categorical variable (BMI) with four levels. Table 2 appears to confirm this but the text on page 10 describes the association only in terms of obesity, the most extreme category. I think this all needs clarifying as at the**

**moment is confusing.**

**Answer:**

We see how our phrasing may be confusing as the BMI categories is an ordinal variable. We highlight that obesity is the outcome of interest both in our Aim (Page 5, Line 100) and in the above-mentioned section (Page 8, Line 151). However, we have now clarified that we are primarily quantifying the association with obesity, but we present all other associations as well (Page 8, Line 153): “Although our aim is to examine obesity, we quantify associations between CS and all categories of BMI as compared to normal weight BMI”

For an extended explanation on the reasoning of choice of model see below.

**Page 7 If the primary outcome is indeed an ordered categorical one why not analyse it as such rather than as unordered?**

**Answer:**

As obesity is the primary outcome of interest (and not the ordered BMI category, see above), we could have approached this in a similar fashion as other studies have done (e.g. Yuan et al. 2016, Reference 15); such studies have performed log-binomial/logistic regressions with a binary outcome (e.g. BMI ≥30). However, by doing this underweight and overweight is included with normal weight in the reference outcome. This could lead to an under or overestimation of the association between CS and obesity, depending on how CS is associated with underweight and overweight BMI. To have normal weight as the reference category, and not exclude other categories, we chose to perform a multinomial logistic regression and only minorly comment on the underweight and overweight category (as these were not primary outcomes). Notably, transition from one level of the outcome to the next higher is not of most interest; the interest is in the development of obesity rather than maintaining a healthy normal weight. Finally, despite our primary interest in obesity and not the ordering of BMI categories, we were ad hoc worried about violations of the proportional odds assumptions (parallel lines assumption) which makes order logit models inappropriate.

Despite this, we see how readers may find a model incorporating the influence of ordering interesting, and that certain meta studies may have an interest in such models. Therefore, we have now performed a generalized ordered logit model (as described by Williams 2016, see below), relaxing assumptions of proportionality, with the same standard error estimator (robust/sandwich) and adjusted for the same factors as our main analysis. As expected, this analysis did not alter our conclusions; the analysis did not differ meaningfully from our original analysis. We have included this in supplementary material (sTable 10), inserted it below this paragraph, and made reference to it in the Method section under Sensitivity Analysis by the following sentence (Page 10, Line 199): “In post-hoc analysis, we examine the influence of CS on ordered BMI categories employing a generalized ordered logit model relaxed of proportionality assumptions (62), …”

We briefly comment on this alternative analysis in the Result section under sensitivity analysis, in addition to other analysis request by other reviewers (Page 14, Line 262): “There was no difference between our main analysis and when excluding those born pre-term, restricting to those born at-term, when not adjusting gestational age (S1 Appendix: sTable 9), or when treating BMI categories as an ordered outcome (S1 Appendix: sTable 10).”

| **Supplementary material excerpt, for reviewer convenience: sTable 10. Crude association and adjusted association using generalized ordered logit estimation.** | | | | | | | |
| --- | --- | --- | --- | --- | --- | --- | --- |
|  | **Crude**  **(n=97,291)** | | |  | **Adjusted^a^**  **(n=97,291)** | | |
|  | **OR** | **95% CI** | ***P*** |  | **OR** | **95% CI** | ***P*** |
| **Underweight to Normal Weight** | | |  |  |  |  |  |
| *Vaginal* | 1 | - | - |  | 1 | - | - |
| *Elective c-section* | 1.15 | 1.00-1.32 | 0.045 |  | 1.13 | 0.98-1.30 | 0.095 |
| *Non-elective c-section* | 1.11 | 0.97-1.27 | 0.126 |  | 1.07 | 0.93-1.23 | 0.331 |
| **Normal Weight to Overweight** | | |  |  |  |  |  |
| *Vaginal* | 1 | - | - |  | 1 | - | - |
| *Elective c-section* | 1.06 | 0.98-1.14 | 0.152 |  | 1.00 | 0.93-1.09 | 0.920 |
| *Non-elective c-section* | 1.10 | 1.02-1.19 | 0.012 |  | 0.99 | 0.91-1.07 | 0.721 |
| **Overweight to Obese** | | |  |  |  |  |  |
| *Vaginal* | 1 | - | - |  | 1 | - | - |
| *Elective c-section* | 1.14 | 1.00-1.31 | 0.057 |  | 1.04 | 0.90-1.20 | 0.571 |
| *Non-elective c-section* | 1.16 | 1.01-1.33 | 0.032 |  | 0.97 | 0.84-1.11 | 0.649 |
| Abbreviations: CI indicates confidence interval, RRR indicates relative risk ratio. Empty cells (-) indicate reference group. | | | | | | | |
| ^a^Adjusted for: Pre-pregnancy maternal BMI, maternal diabetes at delivery, maternal hypertension at delivery, maternal smoking, parity, parental education, maternal age at delivery, birth weight standardized according to gestational age, preeclampsia and gestational age. | | | | | | | |

Reference:

Williams, R. (2016). Understanding and interpreting generalized ordered logit models. *The Journal of Mathematical Sociology*, *40*(1), 7-20. https://doi.org/10.1080/0022250X.2015.1112384

**Page 7 I am not sure I would have said parity was a continuous variable but in any event it was treated as categorical anyway.**

**Answer:** We acknowledge that it might not be suitable to address parity as a continuous variable, we have now removed “continuous” and only included “treated as categorical” in the Method section under Confounders (Page 8, line 166).

**Page 8 For variables which are confounders and whose association is not of primary interest it seems a good idea not to insist on linearity as mentioned here. Why then only relax this for some variables? Why not use splines which make fewer assumptions about the exact shape?**

**Answer:**

We appreciate this comment as also we believe that relaxing the linearity of confounders is important. We initially only relaxed maternal age and maternal pre-pregnancy BMI as we trusted these to be the strongest confounders, and because these factors had been relaxed in a previous influential study on CS and obesity (Yuan et al. 2016. reference 15). We have now kept our original cubic approach, as this may be informative in direct comparison with previous literature, but also included a model where we relax all continuous covariates from our main analysis (Birth weight standardized by gestational age, gestational age, maternal pre-pregnancy BMI and maternal age) using restricted cubic splines with 5 knots at the: 5 27.5 50 72.5 95 percentiles. The more extensive approach using restricted cubic splines did not alter our conclusions, which we now highlight in the Result section under Sensitivity analysis (Page 14, Line 247). We have included this model in our preexisting sensitivity analysis sTable 3 (Supplementary material: Page 5, Line 36) and described our procedure in the Method section under Sensitivity analysis by the following sentence (Page 10, Line 200): “… and further relaxed linearity of all continuous factors (maternal pre-pregnancy BMI, maternal age, birth weight standardized according to gestational age, and gestational age) using restricted cubic splines with five knots at Harrell’s recommended positions (63): 5 27.5 50 72.5 95 percentiles…”

**Page 10 If the authors used logistic regression then the estimates are presumably odds ratios rather than risk ratios which would have required a log-binomial model.**

**Answer:** Using a conventional logistic regression we agree that we would have obtained the odds ratio and using a log-binomial model we would have estimated the risk ratio (or relative risk). However, in STATA using a multinomial logistic regression the exponentiated coefficients are relative risk ratios. To describe these as odds ratio may be misleading, both because this is not correct, and because it might imply that repeated logistic regressions were employed.

We refer to the STATA base manual version 15 Page 1653 for examples on why the multinomial logit (mlogit function) estimates the relative risk ratio, rather than the odds ratio: StataCorp. 2017. Stata 15 Base Reference Manual. College Station, TX: Stata Press. Available at: <https://www.surveydesign.com.au/docs/manuals/stata15/r.pdf>

**References It might be a kindness to the reader to give translated titles for the ones in Swedish although I suppose if you cannot understand the title you are unlikely to try to fetch the article.**

**Answer:** We are thankful to the reviewer for noting this. We have now provided a translated title for reference 2, 27 and 34 (Page 19, Line 385 & Page 21, Line 445 & Line 462), which were originally only presented in Swedish.

**Supplement This finished with eight blank pages. I suspect there was nothing missing.**

**Answer:** We do not note any missing pages in the submitted material. We believe this may be an artifact of file conversion.

**Reviewer #2:**

**Ahlqvist et al. present a retrospective cohort study on the association between birth by Caesarean section and obesity in young adulthood among 97,000 Swedish males. I have detailed my comments below.**

**Major comments:**

**I do believe that replication in science is good and necessary, and research does not have to be novel to be publication-worthy. However, for the association between CS and offspring obesity, there have been around 100 studies (28 studies in Kuhle et al. 2015, 35 studies in Darmasseelane et al. 2014, and about 40 primary studies published since) published to date that looked at the issue from a lot of different angles in a multitude of settings using different kinds of analyses; the majority of studies adjusted for the key confounder maternal pre-pregnancy weight and still found an association. Based on the available evidence, I think it is safe to say that there is a true association. The paper by Ahlqvist et al, while solid, does not offer anything groundbreakingly new that would make me question the presence of that association between CS and obesity.**

**Answer:**

We are thankful for this comment and we concur with the reviser that there have been several previous studies on CS and obesity. However, as we note in the Introduction (Page 4, Line 87), to the best of our knowledge only few studies have differentiated between elective and non-elective CS with regards to obesity. In fact, all systematic reviews we are aware of have not included such analysis or have been outspokenly uncertain regarding the conclusion from such differentiation.

Specifically, Darmasseelane et al. 2014 (Reference 13) describe that there is so few studies (3 studies in their analysis, n=270 in-labour and n=252 pre-labour) differentiating between in-labour-CS and pre-labour-CS that they are unable to make reliable conclusions regarding the impact of type of CS on obesity (See Page 12 Darmasseelane et al. 2014). Although very few studies contributing to this analysis, they found no significant association between emergency/in-labour-CS (Unadjusted OR 1.26, CI 95%: 0.78-2.05, *p*=0.35, i^2^ = 39%) nor elective/pre-labour-CS (Unadjusted OR 1.13, CI 95%: 0.80-1.59, *p*=0.50, i^2^ = 0%) and obesity. The systematic review by Kuhle et al. 2015 (Reference 16) did not consider indication for CS nor type of CS, although they make reference to Darmasseelane (See page 301). Li et al. 2013 (Reference 14) did not consider type of CS in their systematic review. Similarly, the most recent and extensive systematic review, published in PLOS Medicine, by Kaeg et al. 2018 (Reference 18) were unable to differentiate between type of CS (See page 12).

As we note in the Discussion (Page 15, Line 276), prior to our study the largest cohort on CS and obesity with the ability to differentiate between types of CS (maternal requested CS vs non-maternal requested CS) is the study conducted by Li et al. 2014 (Reference 64). Despite incomparability because Li et al. include only children (Mean age 4.9 years), Li et al. observe a substantial amount of CS (n=38,700). However, they are limited to only 1,812 CS on maternal request of which only 145 develop overweight or obesity and do not account for maternal comorbidities nor maternal smoking.

In addition to the limited ability to differentiate between types of CS and the lack of adjustment for important confounders, Darmasseelane et al. meta-analysis of pooled CS and obesity included only 2,975 CS (11 studies) which constitutes 36% of our fully adjusted pooled CS sample. One could argue that it is possible that the lack of adjustment for important confounders and limited sample size may fully explain the association Darmasseelane et al. observed in their pooled analysis (Unadjusted OR 1.22, CI 95%: 1.05-1.42, *p*=0.01, i^2^=22%). Similarly, Kuhle et al. 2015 (Reference 16) which analyzed younger children (Median age 6, range 2-15 years) from 19 studies included a total sample of 33,987 deliveries (i.e. vaginal and CS). Although larger, Kaeg et al. 2018 (Reference 18) include only 5 studies on pooled CS and obesity in adulthood (20-28 y/o), totaling 7,759 CS.

In conclusion, as we also state in the Discussion (Page 15, Line 269), we have presented the so far largest and most comprehensive differentiation between elective and non-elective CS. We believe, as also highlighted by another reviewer, that high-quality studies with large samples are scarce on CS and obesity, and as such the current evidence will misinform clinicians and policymakers alike.

**I have reviewed a number of manuscripts on the subject, and most papers, like the one under review, justify their existence with the claim that confounding has not been sufficiently considered in previous studies (not true), only to go on to either use the same set of confounders as most previous studies, or add one confounder to their models and remove another. Ahlqvist et al perform a sibling-analysis to account for unmeasured familial confounding (as has been done before for this association), but this type of analysis adds confounding by non-shared confounders, as the paper by Frisell et al. 2012 (reference 57 in the manuscript) points out. This bias is not acknowledged or discussed by the authors.**

**Answer:** We appreciate this feedback and we agree that there are limitations with sibling-analysis, as have been described by Frisell et al. 2012 and by others. Although being a powerful tool to address unmeasured shared familial confounding, sibling-analysis may under certain circumstances suffer from larger bias and/or attenuation than conventional analysis. Hence, sibling-analysis should be interpreted with caution and not be interpreted as the main analysis, but rather consider a supplement to conventional analysis with its own limitations and strengths. We are aware that previous studies have applied sibling-analysis in studying CS and obesity/BMI (Yuan et al. 2016, Reference 15 and Rifas-Shiman et al. 2018, Reference 22). However, we are unaware of any previous study employing sibling-analysis when studying type of CS and obesity.

To highlight that we agree with the concerns raised by the reviewer regarding sibling-analysis we have now included a section on this in the Discussion under Strength and Limitations (Page 17, Line 343): “Fifth, despite the ability to control for unmeasured confounding sibling-analysis have several limitations, such as increased bias from non-shared confounders and increased attenuation from measurement error as compared to conventional analysis (57). However, our collection of CS by medical records and standardized measurement of BMI reduces the impact of measurement error. Further, by accounting for observed non-shared confounders we limit any additional confounding to non-shared unobserved confounders. Despite this, caution is warranted when directly interpreting the estimates from sibling-analysis.”

**The other claim to (near-)novelty that the authors make is that "only a few studies [3 studies are referenced] have investigated effects of elective and non-elective CS in separate". I cannot verify or refute that claim, but in the presence of approximately 100 studies on the association, the authors need to demonstrate how they arrived at this number - did they do a systematic review of the literature? The authors also should describe clearly what the definition of elective and non-elective CS is, and if that definition is used consistently in the database.**

**Answer:** We are thankful for this comment, we realize that the sentence is phrased so that it may imply that we have conducted a systematic review of all available literature. We have now rephrased the sentence in the Introduction to the following (Page 4, Line 87): “To the best of our knowledge, only a few studies have investigated effects of elective and non-elective CS in separate (23-25).”

We have now included a definition of elective and non-elective cesarean section in the Method section under Exposure - Mode of delivery (Page 8, Line 142): “In accordance with the reporting standard in the MBR, we define elective CS as pre-labor CS and non-elective CS as CS after the onset of labor.”

Finally, we have now clarified in the Method section under Exposure - Mode of delivery that the reporting procedure was consistent during the period of study (Page 8, Line 143): “There was no alteration in the reporting procedure of elective or non-elective in the MBR during the period of study.”

**Another issue is the external validity of the study, since the men in the study were born more than 30 years ago (1982 to 1987). As expected, the CS rate in the sample is very low at 8.5% and the obesity prevalence sits at 4.9%. Both prevalences are considerably lower than what we see today. With the CS rate between 20 and 30% in most Western countries, I would question whether the population of women who had an elective CS in the 1980s is in any way comparable to women undergoing elective CS these days. The authors try to claim external validity by stating "In addition to aforementioned discrepancies between our study and previous studies, it should be noted that our cohort was born between 1982 and 1987, which could have implications for the comparability to more recent birth cohorts (24). However, several studies conducted on younger children with more recent birth years have suggested similar null associations as those we observed in our cohort (22, 23, 25)." I**

**disagree with that logic: Because more recent studies also had null findings, the current study has external validity?**

**Answer:** We agree, as you also note that we do, that the fact that our cohort was born in the 1980s may influence the generalizability. We are not suggesting that other studies conducted using more recent birth cohort with similar null-findings give us external validity. We merely note that null associations are not unique to older cohorts. Regardless of temporal space of certain cohorts, the negative implications for generalizability and external validity should be concerned with one question: What would make CS unique now, as compared to historically? It may be possible that changes in clinical routines make modern CS associated with risks, which it was not historically. However, the contrary seems more plausible; improvement in clinical routines should make CS (assuming successful improvement) less hazardous in modern cohort as compared to our cohort. Further, rapid increases in obesity worldwide, for which CS often is indicated, may be more present in more recent cohorts. This would induce a greater prevalence of a strong confounder in more recent cohorts, as compared to ours.

Despite this, and in accordance with a other reviewer, we now highlight that the generalizability of our findings may be limited to countries with similar rates of CS as in Sweden during the study period in the Discussion under Strengths and limitations (Page 18, Line 351): “The generalizability of our study may be limited to countries with similar rates of CS as in Sweden during the study period.”

**Minor comments:**

**- Use abbreviation CS consistently throughout**

**Answer:** We are thankful for this feedback and have now corrected this throughout the text in the manuscript (Page 4, Line 91 & Page 14, Line 259).

**- Line 60: "in the offspring" not "in offspring's"**

**Answer:** We have now corrected the above-mentioned typo (Page 4, Line 78).

**- Line 72: "which could inflate the risk for obesity in the non-elective cesarean section" should read "which could inflate the risk for obesity in the non-elective CS group" or "which could inflate the risk for obesity in children born by non-elective CS"**

**Answer:** Thank you for noting this incorrect formulation. We have now rephrased the sentence in the Introduction (Page 4, Line 90) : “…which could inflate the risk for obesity in the non-elective CS group…”

**- Lines 189, 204, 231, 252: Replace "&" with "and"**

**Answer:** We have now removed “&” and replaced it with “and” at the above-mentioned locations (Page 11, Line 229 & Page 14, Line 254 & Page 16, Line 281 & Page 16, Line 302).

**- I would probably forego the adjustment for gestational age as it may be a collider. See the paper by Wilcox et al. 2011 (**[**https://eur01.safelinks.protection.outlook.com/?url=https%3A%2F%2Fwww.ncbi.nlm.nih.gov%2Fpubmed%2F21946386&amp;data=02%7C01%7Cdaniel.berglind%40ki.se%7C14ec1e8131b6495dfab608d745f39c27%7Cbff7eef1cf4b4f32be3da1dda043c05d%7C0%7C0%7C637054785399004990&amp;sdata=vBCyMp45ZmfR1uqApNaZVmhlC8zqTfvv8k4BEnHWnHA%3D&amp;reserved=0**](https://eur01.safelinks.protection.outlook.com/?url=https%3A%2F%2Fwww.ncbi.nlm.nih.gov%2Fpubmed%2F21946386&amp;data=02%7C01%7Cdaniel.berglind%40ki.se%7C14ec1e8131b6495dfab608d745f39c27%7Cbff7eef1cf4b4f32be3da1dda043c05d%7C0%7C0%7C637054785399004990&amp;sdata=vBCyMp45ZmfR1uqApNaZVmhlC8zqTfvv8k4BEnHWnHA%3D&amp;reserved=0)**)**

We agree with the reviewer, and Wilcox et al. 2014, that adjustment for gestational age may induce collider bias (alt. collider stratification) under certain causal pathways. We have now addressed such concerns by leaving out adjustment for gestational age. We now present a model unadjusted for gestational age in the Supplementary material sTable 9 (Page 9, Line 70). There was no difference between our main model and a model not adjusted for gestational age.

As we have conducted above specified analysis, we now describe this in the Method section under Sensitivity analysis (Page 11, Line 207): “Finally, as gestational age may act as a collider under certain causal pathways, we repeated our main analysis excluding adjustment for gestational age.”

**- Line 158-159: "First, a model where we introduced a cubic transformation of maternal age and maternal BMI, to relax the assumption of linearity." Transforming age and BMI doesn't "relax" the assumption, the assumption is always there and should be met for the analysis. I would suggest examining linearity with LOESS or GAM first and then transform (or not) in the main analysis and skipping that part of the sensitivity analysis.**

We acknowledge that by the phrasing of the sentence it seems as we have cubically transformed the scale of the original variable, which does not relax linearity. To clarify, we did not transform the scale of original variable, but introduced a cubic transformation of the specific variable in addition to the original variable. Thereby estimating a coefficient that is equal to the, in addition to linear influence, cubic influence of said variable. We have now clarified this by the following sentence in the Methods under Sensitivity Analysis (Page 9, Line 184): “First, a model where we introduced a cubic transformation of maternal age and maternal BMI, in addition to the untransformed factor, to relax the assumption of linearity”

Further, in accordance with a suggestion from the statistical review we have relaxed all continuous factors in our adjusted model using restricted cubic splines with 5 knots at Harrell’s recommendations, now presented in sTable 3 (Supplementary material: Page 5, Line 36).

**- Line 186: "In our primary analysis, there was an insignificant association between elective CS and obesity [...]" There is no "insignificant" association. Should read: "The association was not statistically significant."**

**Answer:** In accordance with this comment and editorial requests, we have now reformulated the sentence to (Page 11, Line 226): “In our primary analysis, there was no significant association between elective CS and obesity (RRR 1.14, CI 95%: 0.99-1.30) …”

**- Lines 223-226: "In contrast to previous research (10, 13, 15, 16), we did not observe any association between CS and young adulthood obesity. This could be explained by previous studies limited ability to adjust for maternal pre-pregnancy BMI (16), small sample sizes (10, 13, 15, 16), and/or inability to differentiate between elective/pre-labor and non-elective/acute CS (10, 16)." The authors should avoid handpicking a few of the many many studies to give the impression that the existing body of research has serious shortcomings.**

**Answer:** We agree that handpicking a few original studies is a serious limitation, as it might depict the existing body of evidence as flawed. Hence, all the studies we refer to in the above-mentioned section are systematic reviews. With the exemption of Yuan et al. 2016. (reference 15) which we especially highlight since we commend Yuan et al. 2016 in the Introduction (Page 4, Line 84) on their careful investigation to control for unmeasured confounding.

**Reviewer #3: Ali Khashan (University College Cork)**

**The present study was performed to examine the association between mode of delivery and the risk of offspring overweight and obesity in early adulthood. The study used data from the Swedish national registers and included males who with data on height and weight measured at conscription. The study results showed no evidence of an association between elective or emergency CS and the risk of overweight or obesity in male offspring with the relative risk ratios very close to one. This is an important study considering the limited evidence on the association between CS and the risk of obesity in young adults. The manuscript is very well written and the fact that the statistical analysis plan was registered before the analysis was performed is a strength in this paper. The lack of an association between CS and risk of overweight or obesity in early adulthood is an important finding for mothers and clinicians. A key limitation is the fact BMI was measured only once while one would**

**have liked more than one measurement over time. The authors may wish to consider the following comments:**

**1)	I am not sure if I missed this, but what was the age of children at the time of weight and height measurement? Considering that conscription was up to age 47, does this mean the outcome was measured at different age points for different persons? If this were the case, then age at the time the outcome was measured should be taken into account in the analysis.**

**Answer:**

We are thankful for this important comment. We acknowledge that we had not included the age of the conscripts in the first version of the manuscript. In accordance with this comment and with editorial request we have now included a clarification of the median age at conscription in the Method section under Study population (Page 6, Line 133): “In total, 97,291 male conscripts, with a median age of 18 years at follow-up (i.e. conscription), remained eligible for analysis and were included in the final analytical sample”.Further, we now also present the median and interquartile of age at conscription in Table 1, for the total cohort and by exposure category (Page 11, Line 224).

We do not account for age at conscription in analysis, primarily as all men during the study period are conscripted at the age of 18, with very few exceptions. Further, we find it difficult to see how age at conscription would influence mode of delivery, thereby not making it a confounder.

**2)	Although the sibling analysis is an important analysis in this type of study, it is usually used to assess potential familial confounding when there is an association. In other words, it is used to determine whether an observed association is potentially causal or due to shared familial confounding. In this study there was no evidence of an association between CS and offspring obesity, therefore the sibling analysis has no added value and in fact it could be misleading as readers may misinterpret the significant result in the sibling analysis. I would remove the analysis from the manuscript and clarify that because there was no evidence of an association in the cohort analyses, the sibling analysis was not performed.**

**Answer:**

We agree with the reviewer that sibling-analysis usually is employed when there is an association, to evaluate the robustness of such an association to unobserved shared confounders. However, we believe that this approach is flawed. This stems from the notion that confounding may, in several plausible scenarios as described by Mehio-Sibai et al. 2005 (https://doi.org/10.1016/j.annepidem.2004.10.004), bias an estimate towards 0 or towards infinity. For instance, a true causal estimate of 1.2 may be underestimated to 1 because a negative (inverse) confounder bias the estimate towards 0.

In our example, a null association between CS and obesity in the conventional analysis may be biased by an unobserved shared confounder shifting a true causal protective effect (e.g. RRR 0.70) towards infinity and could also bias a true causal risk (e.g. RRR 1.3) towards 0. In conclusion, a null association in conventional analysis does not reduce the necessity to attempt to address unobserved confounders, as a null association may be a result of confounding in either direction.

Despite this, we agree with the comment that readers could be misled to interpret statistically significant results in the categorically discordant sibling analysis. To address ours and your concern that a reader may be misled, we elaborate on this in the Discussion under Comparison with previous research (Page 16, Line 309): “Notably, we observed an association between non-elective CS and overweight in our multinomial discordant sibling sample. However, this may be explained by the small number of exposed cases and the possibility of residual confounding. Hence, we suspected there to be an association between non-elective CS and overweight as a function of confounding by medical indication for CS (23). Indeed, if there is a causal effect of CS on overweight/obesity an association should be present in elective CS as well (23).”

**3)	Although I understand the authors' approach of excluding persons with missing outcome data, excluding persons with missing maternal BMI and missing maternal smoking is not justified. I would include those persons in the analysis and use a missing data indicator for those two variables. Including those persons in the crude analysis would help the authors assess whether their assumption on missing data is accurate or not.**

**Answer:**

We are thankful for this comment. We argue that the missing in Swedish population registers are likely to be Missing Completely At Random (MCAR) or in very few cases Missing Not at Random (MNAR) (Page 6, Line 129). In the scenario of MCAR our complete case analysis will be inefficient but unbiased and in the scenario of MNAR there is no suitable imputation approach available (to the best of our knowledge). We did not present any comparison of a model including those with available CS and BMI without covariate information and our crude complete case model (no missing in any factor). The limitation with such a comparison is that, although it might describe MCAR for covariates, they are unable to investigate the influence of missing in exposure and outcome. For reviewer interest we have included such an analysis below. However, we choose not to include such analysis in the manuscript given its limitation, and that we are concerned that readers may be led to believe that such analysis providence evidence that MCAR holds (not true).

For adjusted analysis, assuming that we incorrectly assume the missing to be MCAR, and it is in Missing At Random (MAR), a missing data indicator would not reduce bias. As describe by Donders et al. (2006) (<https://doi.org/10.1016/j.jclinepi.2006.01.014>), the missing data indicator approach in non-randomized settings will yield biased estimates both under MCAR and under MAR.

Given that missing indicator variable bias both under MAR and MCAR, we do not see how readers could compare an adjusted model using the missing indicator variable approach (as suggested) with our adjusted complete case analysis to disentangle if our assumption on missing data is accurate. It is fully possible, that even under MCAR (which will not bias complete case), a statistically significant association is present in missing indicator variable approach but not in complete case analysis. This would lead to the readers being misled to believe that there is a significant association, when this is merely a function of bias in the missing indicator approach in non-randomized settings.

| **Partial missing analysis, comparing the complete case model and a model including those individuals with missing data on covariates.** | | | | | | | | | | | |
| --- | --- | --- | --- | --- | --- | --- | --- | --- | --- | --- | --- |
|  | **Crude complete case (n=97,291)** | | | |  | **Crude with missing (n=153,117)** | | | |  | **Difference in RRR between models** |
|  | **Cases, No. (%)** | **RRR** | **95% CI** | ***P*** |  | **Cases, No. (%)** | **RRR** | **95% CI** | ***P*** |  | ***P*** |
| **Underweight vs Normal Weight** | | | |  |  |  |  |  |  |  |  |
| *Vaginal* | 5,491 (6.2) | 1 | - | - |  | 8,499 (6.1) | 1 | - | - |  | - |
| *Elective cesarean section* | 224 (5.4) | 0.88 | 0.76-1.01 | 0.064 |  | 370 (5.4) | 0.89 | 0.80-0.99 | 0.033 |  | 0.758 |
| *Non-elective cesarean section* | 230 (5.6) | 0.92 | 0.80-1.05 | 0.22 |  | 426 (6.0) | 0.99 | 0.89-1.10 | 0.840 |  | 0.110 |
| **Overweight vs Normal Weight** | | | |  |  |  |  |  |  |  |  |
| *Vaginal* | 13,720 (15.4) | 1 | - | - |  | 21,332 (15.3) | 1 | - | - |  | - |
| *Elective cesarean section* | 650 (15.7) | 1.02 | 0.93-1.11 | 0.669 |  | 1,047 (15.4) | 1.00 | 0.94-1.07 | 0.934 |  | 0.557 |
| *Non-elective cesarean section* | 671 (16.3) | 1.07 | 0.98-1.17 | 0.113 |  | 1,116 (15.6) | 1.03 | 0.97-1.10 | 0.338 |  | 0.183 |
| **Obese vs Normal Weight** | | | |  |  |  |  |  |  |  |  |
| *Vaginal* | 4,335 (4.9) | 1 | - | - |  | 6,913 (5.0) | 1 | - | - |  | - |
| *Elective cesarean section* | 229 (5.5) | 1.14 | 0.99-1.30 | 0.069 |  | 370 (5.4) | 1.09 | 0.98-1.22 | 0.105 |  | 0.375 |
| *Non-elective cesarean section* | 231 (5.6) | 1.17 | 1.02-1.34 | 0.027 |  | 410 (5.7) | 1.17 | 1.06-1.30 | 0.003 |  | 0.956 |
| Abbreviations: CI indicates confidence interval, RRR indicates relative risk ratio. Empty cells (-) indicate reference group. | | | | | | | | | | | |

**4)	The authors highlight the potential misclassification of CS in preterm births, have they considered performing an analysis excluding preterm birth to check whether this potential misclassification is an issue? This is an analysis worth performing.**

**Answer:** We are very thankful for this suggestion. We agree that this may provide some insight into the influence of potential misclassification, as such we have now included a sensitivity analysis where we exclude those born prior to 37 full weeks of gestation and restrict our analysis to those born at-term (≥37 & <42). We conclude that this sensitivity analysis does not alter our conclusion. We have now described this analysis in the Method section under Sensitivity analysis (Page 11, Line 203): “As previous validity reports (32) have noted that there may be some misclassification of the type of CS in the MBR and that this misclassification is primarily present in pre-term deliveries which were misclassified as elective, while being non-elective, we repeated our main analysis excluding those born pre-term (<37 full weeks of gestation) and repeated our main analysis restricted to those born at-term (≥37 and <42 weeks of gestation).”

We conclude in the Results under Sensitivity analysis (Page 14, Line 262): “There was no difference between our main analysis and when excluding those born pre-term, restricting to those born at-term, …”

Finally, as we now have addressed our previously raised concerns about misclassification, we have now altered the specific section in the Discussion under Strengths and limitations describing this (Page 17, Line 337): “Further, there may be some misclassification of non-elective CS among pre-term deliveries (32). However, our analysis excluding pre-term deliveries and restricting to at-term deliveries was consistent with our main analysis, suggesting that this misclassification of non-elective CS does not influence our conclusion. Despite this, a certain proportion of misclassification is expected and could inflate the risk of an offspring developing obesity in the elective CS group, if an offspring of a non-elective CS is at higher risk of developing obesity as compared to those with a true elective CS, given medical indications.”

**5)	This study adds to a body of evidence from Sweden, that we and others did, showing limited evidence, if any, of an association between CS and asthma, type 1 diabetes, autism, ADHD and psychosis and the authors cited our two recent papers on CS and child obesity (reference 23 and 25) and we have two papers in press showing almost similar results from New Zealand and UK cohorts. The authors may want to have a more detailed discussion of these negative findings in terms of clinical practice and the fact CS does not seem to cause child morbidity, when good quality data and robust statistical analyses are used. Another discussion point is the fact Sweden has a low CS rate compared to other high income countries. Is it possible that elective CS in Sweden is elective due to medical indications and that what causes child morbidity, including obesity, is the unnecessary elective CS? I doubt this is the case, however, it is worth discussing the generalisability of results from a**

**country with low CS rate to countries with high CS rate.**

**Answer:**

We are greatly appreciative of the work conducted by the reviewer and we concur that most studies with higher quality seem to provide limited support for associations between CS and subsequent morbidity. However, as noted by other reviewers and editors, our study and a few high-quality observational studies may be insufficient evidence to directly address clinical practice. Although we are confident in our findings and the reviewer’s studies, we warrant caution in directly translating observational findings into clinical practice. Despite the need for caution, we conclude “This suggests that there is no clinically relevant association between CS and the development of obesity” (Page 18, Line 357).

We agree that it is interesting to further study unnecessary (not medically warranted) elective CS. To highlight this and encourage future studies to perform in-depth investigation into unnecessary CS we have now included a section addressing this (Page 16, Line 315): “Despite unnecessary CS being subject to some criticism (69), it is of interest to further study unnecessary CS and its possible association to offspring morbidity (70). Although Swedish registers enable us to differentiate between elective and non-elective, they do not enable us to perform detailed classifications of CS using such classifications as the Robson Classification (71), which may provide further insight into the potential role of CS in offspring health.”

We agree that the prevalence of CS is low in Sweden as compared to other high-income countries. It is not clear how this impacts the generalizability. For instance, it may be that this reduces confounding in our cohort, or it may be that confounding is more present in our cohort. It is unlikely that the exposure itself has changed dramatically since the mid 1980s; the medical procedure of CS is largely similar although certain routines have been modernized (such as regional anesthesia over general anesthesia). We have now highlighted this as a limitation in the Discussion under Strengths and limitations (Page 17, Line 351): “The generalizability of our study may be limited to countries with similar rates of CS as in Sweden during the study period.”
