# Supplementary material for: Elective and nonelective cesarean section and obesity among young adult male offspring: A Swedish population–based cohort study
Source: PLoS Med. 2019 Dec 6;16(12):e1002996. doi: 10.1371/journal.pmed.1002996 (PMC6897402; doi:10.1371/journal.pmed.1002996)
Supplement: Attachment — Submitted filename: Acceptence Point-by-point Addressal.docx [file pmed.1002996.s014.docx]

**POINT-BY-POINT ADDRESSAL OF COMMENTS
-----------------------------------------------------------**

**Requests from the editors:**
**- I'd suggest removing "the association with" from the title, as there is no association ("... C section and obesity among young adult male offspring ..."). Apologies for making requested changes to the title again.**

**Answer:**

We acknowledge that it is more suitable to phrase the title to accurately depict that we did not find evidence of any association. The title now reads: ”Elective and non-elective cesarean section and obesity among young adult male offspring: a Swedish population-based cohort study”  **- Please add some summary demographic details for the conscripts in the abstract?**

**Answer:** We have now added a sentence including the, in our view, most relevant demographic information in the abstract: “The mothers of the conscripts were on average 28.5 (standard deviation 4.9) years old at delivery, had a pre-pregnancy BMI of 21.9 (standard deviation 3.0) and 41.5% of the conscripts had at least one parent with university level education.” **- I think the Author Summary could benefit from some "what do our findings mean" points and could mention the need to study young female offspring, for example?**

**Answer:** We were initially very brief in the “what do our findings mean” section. We have now included two additional points:

- “Mode of delivery may not be an important factor in the origins of overweight and obesity.
- Cesarean section may not serve a role in the obesity epidemic, and as such should not be a target for intervention when attempting to reduce the burden of obesity.
- Future research should include female offspring when examining the role of cesarean section and obesity, to critically evaluate any sex-specific role of cesarean section in obesity.”

**-Please use square brackets in the main text for references**

**Answer:** We have now reformatted the referencing style to Vancouver using square brackets. As per production requirements, we have also removed spacing between multiple references.

**Production Issues:**

**FIGURES and GRAPHICS:**

**1. Please remove embedded figures from your manuscript file and ensure the most up to date versions of your figures are uploaded to our submission system as separate TIF or EPS files. Please do not remove the figure legends and captions.**

**Answer:** We have now removed the embedded figure and uploaded it as separate TIF files formatted using PACE software.

**2. Please ensure that all main figure files are cited in ascending numerical order in the main text of the article.**

**Answer:** The figure is cited as the first and only figure.

**3. Please ensure that all main figures are referenced in the manuscript as Fig 1, Fig 2, etc. (including capitalization), rather than Figure 1, Figure 2, etc. Please note, however, that the file names themselves must not include the space, i.e. Fig1.tif, Fig2.tif, etc.**

**Answer:** The figure file is cited as “Fig 1” and file name have now been changed to not include a space (Fig1.tif).

**4. Please ensure that all main figure legends are placed after the paragraph containing their first in-text citation.**

**Answer:** The figure legend is placed after the paragraph containing its first in-text citation.

**TABLES and BOXES:**

**1. Please cite main tables in ascending numerical order in your main article file.**

**Answer:** All the main tables and supplementary tables are cited in ascending numerical order in the main article file.

**2. Please ensure that all main tables in their entirety (including titles and footnotes), are placed after the paragraph containing their first in-text citation.**

**Answer:** We have placed all main tables after the paragraph containing their first in-text citation. Please note, Table 2 is put on a separate page, directly following the paragraph containing its first citation, as it is constructed using a landscape format.

**3. Table 1 uses returns to control text wrapping. This is indicated by a [¶ or ↵] symbol when "Show Paragraph Marks" is turned on in Word. Please either split those cells into separate cells to achieve alignment or remove the returns.**

**Answer:** We have removed all returns in Table 1.

**METADATA:**

**1. Please ensure that the CRediT author contributions listed for each co-author are completed in full. To qualify for authorship, each author must meet at least one of the seven core contributions (conceptualization; data curation; formal analysis; investigation; methodology; software; validation), as well as at least one of the writing contributions (original draft preparation; review and editing). Authors may also be noted against any of the remaining contributions, however these alone will not qualify a contributor for authorship.**

**Author Margareta Persson is noted as contributing to "Writing - review and editing" only, which, as mentioned above, does not qualify them for authorship. Please ensure that the full contributions of each author are acknowledged in the "Add/Edit/Remove Authors" section of our submission form. If they do not meet criteria for authorship please let us know as soon as possible by emailing** [**plosmedicine@plos.org**](mailto:plosmedicine@plos.org)**.**

**Answer:** We have now clarified that Margareta Persson qualifies for authorship as she, in addition to writing, contributed to Methodology.

**2. In accordance with PLOS' data policy, please ensure that your Data Availability Statement in the submission form clearly identifies how readers can access your data. Data cannot be available on request, or only accessible by contacting one of the authors. Note that your Data Availability Statement will be typeset as it is written, so please ensure it is in complete sentences and appears as you would like it to in the published version.**

**For details on our data policy, please see** [**https://eur01.safelinks.protection.outlook.com/?url=http%3A%2F%2Fjournals.plos.org%2Fplosmedicine%2Fs%2Fdata-availability&amp;data=02%7C01%7Cdaniel.berglind%40ki.se%7Ca3b04bed87ec477917bc08d75e15ac6b%7Cbff7eef1cf4b4f32be3da1dda043c05d%7C0%7C0%7C637081319492074914&amp;sdata=MuV42%2Fr%2BeISzsWlNL8MfLFBCJ%2FPqemDDmhR7VMHICS0%3D&amp;reserved=0**](https://eur01.safelinks.protection.outlook.com/?url=http%3A%2F%2Fjournals.plos.org%2Fplosmedicine%2Fs%2Fdata-availability&amp;data=02%7C01%7Cdaniel.berglind%40ki.se%7Ca3b04bed87ec477917bc08d75e15ac6b%7Cbff7eef1cf4b4f32be3da1dda043c05d%7C0%7C0%7C637081319492074914&amp;sdata=MuV42%2Fr%2BeISzsWlNL8MfLFBCJ%2FPqemDDmhR7VMHICS0%3D&amp;reserved=0)

**Answer:** As we outline, Swedish law prohibits us from sharing data. Yet, readers can acquire data through a process outlined in the link provided in the current statement.

**ARTICLE FILE:**

**1. Please indicate the corresponding author in the byline by placing an asterisk (*) after their affiliation number.**

**Answer:** We have now included an asterisk after Dr. Berglind's affiliation number.

**2. Please include the corresponding author's email address on the title page of your manuscript, indicated by an asterisk (*). Only an asterisk and the email address itself are required.**

**Answer:** We now include an asterisk followed by Dr. Berglind’s email.

**3. Please remove author degree titles from the byline of your article file.**

**Answer:** We have now removed all degree titles from the byline of our article file.

**4. Please remove the funding information from your manuscript file. The published version is pulled automatically from the “Financial Disclosure” field in the submission form and must not be duplicated. Please therefore take this opportunity to check that the information in this field is correct and completed in full.**

**Answer:** We have now removed the funding information from our manuscript file.

**5. Please remove the Competing Interests section from your manuscript file. The published version is pulled automatically from the "Competing Interests" field in the submission form and must not be duplicated. Please therefore take this opportunity to check that the information in this field is correct and completed in full.**

**Answer:** We have now removed the competing interest section from our manuscript file.

**6. Please remove your Author Contributions section from your manuscript file as this information is automatically pulled from the authors section of our submission form and must not be duplicated. Please therefore take this opportunity to check that the information in this field is correct and completed in full.**

**Answer:** We have now removed the Author Contributions from our manuscript file.

**7. Please remove the data availability information from your manuscript file as this is automatically pulled from the "Data Availability" field in the submission form and must not be duplicated. Please therefore take this opportunity to check that the information in this field is correct and completed in full.**

**Answer:** We have now removed the Data Availability statement from our manuscript file.

**8. Please ensure that your Ethics statement is available in the Methods section of your manuscript in its entirety.**

**Answer:** We have now moved our ethics statements and placed it into the method as the final paragraph.

**REFERENCES:**

**1. Please put all reference citations in square brackets separated by either commas or dashes with no spaces, e.g. [1,2,3] or [1-3].**

**Answer:** We have now changed the referencing style to Vancouver using brackets without spacing.

**2. Please make sure all references are cited in ascending numerical order in the text.**

**Answer:** We have ascertained that all the references are cited in ascending numerical order.

**SUPPORTING INFORMATION:**

**1. Please upload your supporting information as individual files, and use the following naming format: S1 Text; S1 Fig; S1 Table, S1 Data, etc. Each file type should start as S1 and increase numerically: S1 Text, S2 Text, etc. Note that all relevant in-text citations and captions will need to be updated accordingly.**

**Answer:** We have now uploaded our supporting information as individual files and edited all in-text citations to “S(number) Table”.

**2. If you'd prefer to keep your supporting information in one file, please edit any citation of embedded figures and/or tables within your Supporting Information files and the main manuscript to use letters rather than numbers, e.g. “Table A in S1 Text” or "Fig B in S1 Appendix.".**

**Answer:** We have now split our Appendix to individual files (see above).

**3. Please ensure that all Supporting Information files use the following format verbatim throughout the manuscript (including legends and all in-text citations): S1 Fig, S1 Table, S1 Text, S1 Data, etc. Each file type should start at 1 and increase numerically. Please note, however, that the file names themselves must use an underscore rather than a space, i.e. S1_Fig.tif, S1_Table.xlsx, S1_Text.doc, S1_Data.xlsx, S1_PRISMA_Checklist.docx, etc.**

**Answer:** We have now ensured that all supporting information files use the appropriate format.

**4. Please ensure that each supporting information file has a legend at the end of the manuscript file, after the Reference list.**

**Answer:** After the Reference list we now state the types and titles of all included supporting information files.

**5. Please note that your submission cannot contain any references to generic "supporting information" files and instead must refer to specific files using our naming convention (S1 Fig, S1 Table, S1 Text, S1 Data, SI PRISMA Checklist, etc.)**

**Answer:** We have ensured that our manuscript does not contain any references to generic supporting information.
